# CPDP: Contrastive Protein–Drug Pre-Training for Novel Drug Discovery

**DOI:** 10.3390/ijms26083761

**Published:** 2025-04-16

**Authors:** Shihan Zhang, Xiaoqi Wang, Fei Li, Shaoliang Peng

**Affiliations:** 1 College of Computer Science and Electronic Engineering, Hunan University, Changsha 410082, China; zhangsh@hnu.edu.cn; 2 School of Computer Science, Northwestern Polytechnical University, Xi’an 710072, China; xqw@nwpu.edu.cn; 3 Computer Network Information Center, Chinese Academy of Sciences, Beijing 100850, China; 4 The State Key Laboratory of Chemo/Biosensing and Chemometrics, Hunan University, Changsha 410082, China

**Keywords:** novel drug discovery, multi-modal learning, cross-domain knowledge integration

## Abstract

Novel drug discovery and repositioning remain critical challenges in biomedical research, requiring accurate prediction of drug–target interactions (DTIs). We propose the CPDP framework, which builds upon existing biomedical representation models and integrates contrastive learning with multi-dimensional representations of proteins and drugs to predict DTIs. By aligning the representation space, CPDP enables GNN-based methods to achieve zero-shot learning capabilities, allowing for accurate predictions of unseen drug data. This approach enhances DTI prediction performance, particularly for novel drugs not included in the BioHNs dataset. Experimental results demonstrate CPDP’s high accuracy and strong generalization ability in predicting novel biological entities while maintaining effectiveness for traditional drug repositioning tasks.

## 1. Introduction

Novel drug discovery is a complex, multi-stage process focused on identifying compounds that effectively treat specific diseases [1,2,3,4]. A key challenge is predicting drug–target interactions (DTIs), which helps determine which molecules are likely to bind biological targets [5,6,7,8,9]. Traditional drug discovery methods, such as molecular simulation and protein structure analysis, rely on physiology-based and target-based approaches [4]. However, these methods are resource-intensive and time-consuming, limiting the rapid development of new therapeutics.

With the advancement of deep learning (DL) and the growing volume of drug-related data [10,11], graph neural network (GNN) based methods have emerged as powerful tools for predicting DTIs by modeling biomedical heterogeneous networks (BioHNs) [12]. These methods excel in drug repositioning and predicting interactions within the network. For instance, DTINet [6] uses unsupervised learning to generate low-dimensional representations for DTI prediction. NeoDTI [7] leverages neighborhood information to learn topology-preserving representations. However, they rely heavily on graph structures, which makes it challenging to predict DTIs for new drugs lacking existing associations in the graph [10]. GEFA [8], which integrates pre-trained protein embeddings with attention mechanisms, enhances DTI prediction but faces a decline in effectiveness when dealing with new targets and drugs. These challenges underscore the limitations of GNN-based methods in the context of new drug discovery.

Other approaches, such as pre-trained large language models (LLMs) like GPT [13], hold promise for application in biomedical research due to their success in zero-shot and few-shot learning [14,15,16,17]. BioGPT [16] is a generative pre-trained transformer for biomedical text generation, while PMC-LLaMA [15], fine-tuned on 4.8 million biomedical papers, enhances medical knowledge and improves performance in the domain. However, they struggle to capture crucial biomedical features, such as molecular structures, protein sequences, and biological pathways, relying mainly on prior labels rather than structured data. Esm-2 [18] is a Transformer-based model that learns evolutionary information from protein sequences by converting amino acid sequences into numerical vectors.

To extend the outstanding DTI prediction capability of GNN-based methods to new drug discovery, we propose the Contrastive Protein–Drug Pre-Training (CPDP) framework. We first integrate data from various databases, including DrugBank (v4.3) [19], the Therapeutic Target Database (TTD) [20], and PharmGKB [21], to construct BioHNs dataset for subsequent experiments. Afterward, CPDP integrates GNN-based network representation methods and biomedical LLMs, to construct a common embedding space through non-linear projection layers. Through contrastive learning, CPDP aligns actual drug-protein target associations in BioHNs. We then validate the DTI prediction performance of CPDP on novel drugs not present in the BioHNs. In summary, our contributions are as follows:

1. We construct a common embedding space through the CPDP framework, which integrates protein and drug representations from various dimensions, thereby enhancing the prediction of DTIs.
2. We employ contrastive learning for representation alignment to address the issue of sparse training data, while also designing weak labels to retain diverse DTI information from BioHNs and mitigate overfitting.
3. CPDP demonstrates strong performance on novel drug discovery and drug repositioning tasks without relying on predefined graph structures, showing superior generalization to unseen biomolecular entities.

## 2. Results and Discussions

In the following subsections, we first focus on CPDP’s ability to simulate novel drug discovery, followed by its application in drug repositioning.

### 2.1. Using CPDP to Simulate Novel Drug Discovery

In the field of novel drug discovery, researchers aim to predict potential interactions for newly discovered or less studied drugs that lack association information, a challenge commonly referred to as the zero-shot problem.

We use the incremental drug data from DrugBank v5.1 [22] compared to DrugBank v4.3 [19], along with their associated DTIs, as the test set. This includes 125 newly introduced drugs and 156 valid DTIs, as detailed in Section 3.1.

To showcase its zero-shot capability, we begin by providing a protein and one known interacting drug, along with *N* randomly chosen non-interacting drugs in each case. This setup simulates real-world research scenarios where the goal is to identify the most promising drug from a set of candidates. We choose advanced LLMs, specifically Llama-7B [23,24], for comparison, as LLMs can be considered zero-shot models without explicit instructions or additional unlabeled data [13].

On the one hand, CPDP filters relevant drugs by calculating association likelihood scores and evaluates prediction performance using Top-k precision. We test CPDP with different protein and molecule representation models. For proteins, we apply MSSL2drug [9], a self-supervised model based on BioHNs. For molecules, we apply JTVAE [25], Llama [23,24], and BioGPT [16], considering both SMILES [26] and natural language representations. This demonstrates CPDP’s ability to generalize network-based DTI prediction methods to unknown drugs without network association data, simulating the process of discovering new drugs.

On the other hand, we directly query Llama-7B [23,24] to identify the potential associated entity from the same candidate drugs. To ensure optimal performance of the LLMs, we employed the following prompts:

Prompt 1: *“Please select one and the most relevant drug for treating or managing <target> from the following options: <drugs>”.*
Prompt 2: *“Please select one and the most relevant drug for treating or managing <target> from the following options: <drugs>. Answer “None” if you cannot select one drug from the list or require more information. You must start by choosing one from the drug’s name or “None””.*

The results are shown in Table 1 and Table 2. MSSL2drug-JTVAE CPDP performs best at both *N* = 4 and *N* = 9, improving performance by 13% to 76% compared to other molecular representations, demonstrating that JTVAE’s high-quality molecular representations enhance CPDP’s generalization. Particularly when *N* = 9 (Table 2), LLama’s performance is unstable due to prompt issues, but CPDP still maintains a strong predictive capability. CPDP’s Top-3 accuracy shows an improvement of 85–129% over the Top-1 accuracy, demonstrating its robustness.

**Figure 1:**
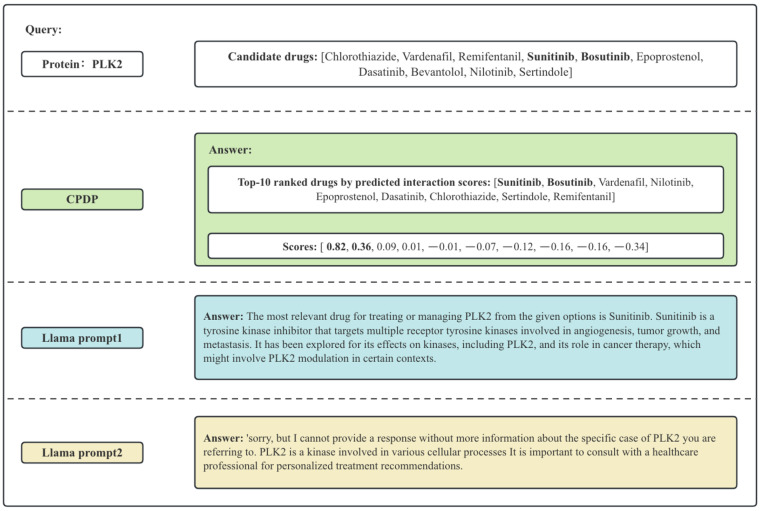
Example of the answer of CPDP and LLama. The bolded drugs are those that actually interact with PLK2.

To better illustrate the differences between CPDP and Llama, we present a specific example, as shown in Figure 1. The scores represent the predicted likelihood of interaction between protein and drug pairs. CPDP can calculate the likelihood scores, allowing the selection of multiple potential drugs in a batch (as shown in Figure 1, Sunitinib and Bosutinib have significantly higher predicted scores compared to other drugs). LLMs typically suggest the most likely drug interaction, but their response quality depends on the prompt, which potentially lead to incomplete or suboptimal recommendations.

Additionally, we list the top 10 cases with the highest predicted association likelihood scores by MSSL2drug-JTVAE CPDP, along with the corresponding literature evidence, as shown in Table 3.

### 2.2. Using CPDP to Simulate Drug Repositioning

We found that CPDP naturally possesses the capability to simulate drug repositioning problems. The core issue lies in identifying potential uses of existing drugs for new disease treatments or novel therapeutic targets.

Following the experimental approach in Section 2.1, we randomly sample 10% of proteins from BioHNs as cold-start protein targets, which were not included in the training process. CPDP then predicts the most relevant drug among randomly selected drugs. For proteins, we apply MSSL2drug [9], Esm-2 [18], Llama [23,24], and BioGPT [16], considering different dimensions of representation methods, including network structure, protein sequences, and natural language. For molecules, we apply JTVAE [25] to represent them from the perspective of SMILES notation, as Section 2.1 shows JTVAE’s strong generalization ability.

The results are shown in Table 4 and Table 5. Considering Top-1 accuracy, Esm2-JTVAE CPDP demonstrates strong stability, with only a 7.6% decrease when the number of irrelevant drugs increases by 1.25 times. Compared to Llama, CPDP outperforms LLMs relying solely on natural language features by 55.9% to 154.6%. This improvement highlights the effectiveness of integrating multi-dimensional representations. In the BioHNs scenario, CPDP leverages existing representation models and an embedding alignment framework to more accurately predict relevant drugs for cold protein targets.

Furthermore, we evaluate CPDP’s drug repositioning capability from another perspective. For each protein, we predict the association likelihood scores with all drugs and calculate AUPR and AUROC as comparison metrics. CPDP is compared with traditional GNN-based representation models, including ZeroBind [37], DeepDTA [38], and deepDTnet [39]. ZeroBind [37], a protein–ligand binding affinity prediction framework, uses a meta-learning approach with strong generalization to quickly adapt to new tasks with limited training samples, making it a few-shot learning method.

**Figure 2:**
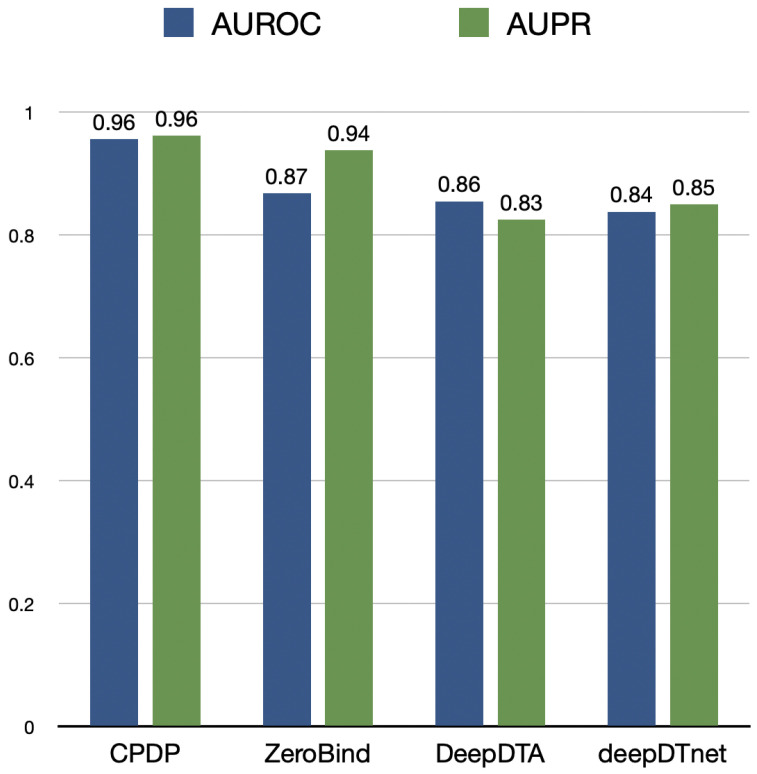
The AUROC and AUPR values of CPDP and other methods evaluated on drug repositioning task.

The experimental results are shown in Figure 2. CPDP achieves an AUROC of 0.96, outperforming ZeroBind by 9.2%. DeepDTA and deepDTnet exhibit relatively weaker performance. CPDP demonstrates a significant advantage in drug repositioning tasks, enabling more accurate drug–target interaction predictions. Although ZeroBind falls short of CPDP in terms of AUROC, its high AUPR indicates strong performance in positive case predictions.

### 2.3. Ablation Experiment

The projection module is essential for mapping and aligning protein and drug representations. To analyze the impact of different projection methods and layer depths, we conducted a series of ablation experiments. Specifically, we examined linear projection and nonlinear projections with 2, 3, 5, and 12 layers. The study aims to determine whether protein or drug representations have a greater impact on CPDP and to evaluate the effects of shallow (linear or two-layer nonlinear) versus deep projections on CPDP’s representation capability.

Predicting molecules that interact with a specific protein can be formulated as a classification task. Given the many-to-many DTIs associations in BioHNs, this task can be further categorized as a multi-label classification problem. To evaluate both the precision and recall of CPDP, we employ *precision@k* and *recall@k*, defined as follows:

(1) $$precision@k=\frac{\left|y^{\left(k\right)}\cap {\hat{y}}^{\left(k\right)}\right|}{\left|{\hat{y}}^{\left(k\right)}\right|}$$

(2) $$recall@k=\frac{\left|y^{\left(k\right)}\cap {\hat{y}}^{\left(k\right)}\right|}{\left|y^{\left(k\right)}\right|}$$

where *y* is the actual label set, $\hat{y}$ is the predicted label set generated by CPDP. The *precision@k* signifies the proportion of correctly predicted labels among the top *k* predicted labels, indicating the accuracy rate, while *recall@k* denotes the proportion of correctly predicted labels among the actual labels, indicating the comprehensiveness rate.

We then calculate the *average precision@k k = 1* (AP) and *average recall@k k = 5* (AR) for each protein to indicate the prediction accuracy of the Top-1 results and comprehensiveness of the Top-5 results, respectively. Following this, we compute the *mean average precision@k* (MAP) and the *mean average recall@k* (MAR) for all proteins as follows:

(3) $$MAP=\frac{1}{m}\sum_{m}^{i=1}AP@k\left(k=1\right)=\frac{1}{m}\sum_{i=1}^{m}\sum_{j=1}^{k=1}precision@j$$

(4) $$MAR=\frac{1}{m}\sum_{m}^{i=1}AR@k\left(k=5\right)=\frac{1}{m}\sum_{i=1}^{m}\sum_{j=1}^{k=5}recall@j$$

where *m* represents the number of proteins. We adopt MAP to reflect the proportion of correct labels in the Top-1 predictions across all target biological entities, measuring the model’s precision. We adopt MAR to reflect the proportion of true labels covered in the Top-5 predictions, evaluating the model’s recall capability.

**Figure 3:**
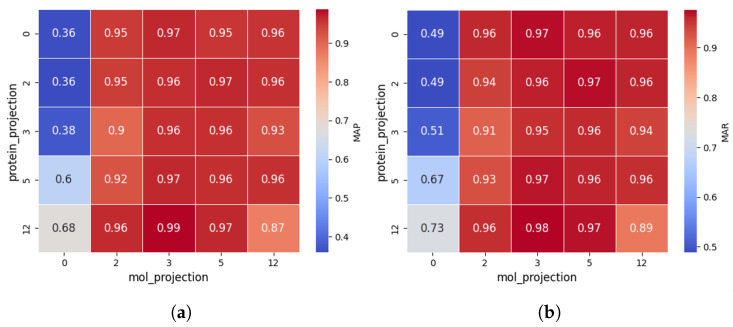
Comparison of MAP (**a**) and MAR (**b**) across different projection layer configurations in CPDP. Zero denotes linear projection and the others represent the corresponding number of non-linear projection layers.

The results are shown in Figure 3.

The impact of protein projection layers is reflected along the vertical axis. When non-linear projections are applied to molecular features (i.e., ≥2 layers), both MAP and MAR consistently remain above 0.9, indicating that protein projection layers have a relatively minor effect. This may be because the initial protein representations are already strong, reducing reliance on additional transformations. However, this does not mean protein projection layers are entirely insignificant. When molecular features are weak (linear projection), increasing protein projection layers boosts MAP and MAR by up to 88.9% and 49.9%, respectively.

The impact of molecular projection layers is reflected along the horizontal axis. When the number of layers is ≥2, MAP rapidly increases to 0.95+, and MAR reaches 0.94+, suggesting that optimizing molecular representations plays a crucial role in CPDP’s predictive performance. This is likely because drug representation is crucial in DTI prediction, as protein structures are relatively stable, while drug molecules vary significantly, requiring more complex modeling to capture their features.

## 3. Materials and Methods

We introduce the CPDP embedding alignment framework for biomedical entities, built on extensive biomedical data and existing biological representation models. Section 3.1 introduces the dataset used in this study. In Section 3.2, we outline the workflow of CPDP, followed by a detailed explanation of the contrastive learning process in CPDP in Section 3.3.

### 3.1. Datasets

Following established cutting-edge methods, we construct BioHNs, a heterogeneous biomedical network dataset, according to deepDTnet [39]. Specifically, BioHNs include object types such as drugs, proteins, and diseases, along with relationship types including DTIs, drug–disease interactions (DDIs) and drug–drug interactions.

More specifically, we collect DTI information from DrugBank database (v4.3) [19], TTD [20], and PharmGKB [21]. For each drug, its chemical structure is extracted from DrugBank [19] in the simplified molecular input line entry system (SMILES) strings [26], while each protein was mapped to its Entrez ID through NCBI [40]. DDIs are attained from several public resources, including repoDB [41], DrugBank(v4.3) [19] and DrugCentral databases [42] by fusing drug indications. We standardized disease names using Medical Subject Headings (MeSH) and Unified Medical Language System (UMLS) vocabularies [43], and then mapped them to MedGen ID based on NCBI [40].

**Figure 4:**
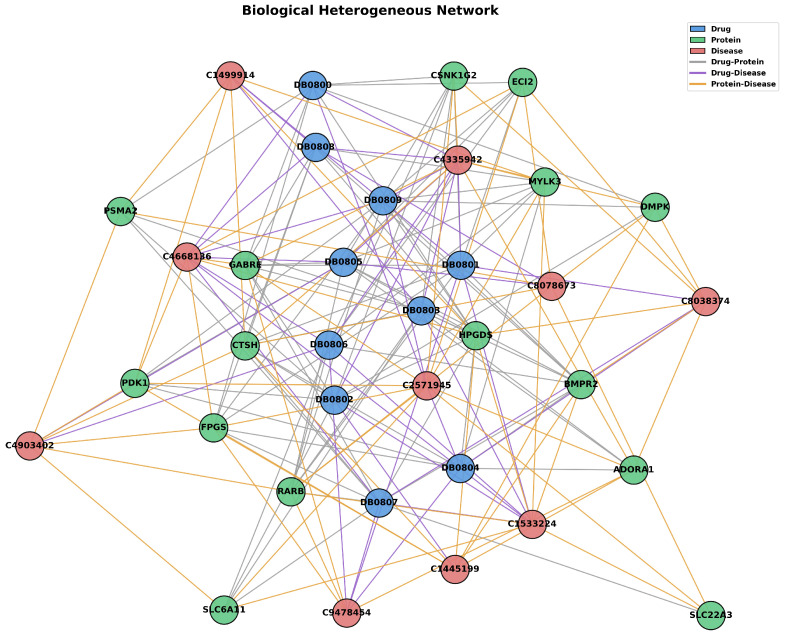
This is a partial schematic of the BioHNs network, showing some of the relationships and structures.

Finally, BioHNs include 670 drugs, 1894 proteins, and 431 diseases, along with detailed interaction data: 4839 DTIs, 1103 DDIs, and 118,364 drug–drug interactions. A sample illustration of BioHNs data is shown in Figure 4.

In the data preprocessing stage, we divide the dataset based on DTIs, with a training-to-validation ratio of 9:1. The test set is sourced from the incremental data of DrugBank v5.1 [22] compared to v4.3 [19], which is entirely unknown to CPDP. We train CPDP using a limited number of DTI labels from BioHNs, with a proportion of 0.004 (≈4839/(670×1894)). The dataset division is shown in Table 6.

### 3.2. Workflow

**Figure 5:**
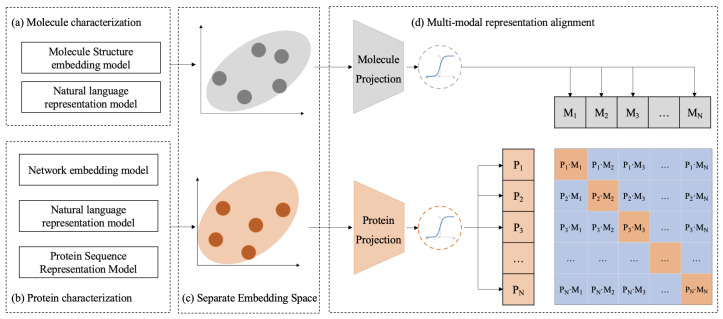
The schematic workflow of CPDP. (**a**,**b**) Protein and molecule representations are derived using different pre-trained models. (**d**) CPDP jointly trains a molecule projection and a protein projection to map their separate embedding space (**c**) into a shared representation space and predict the association scores for a batch of (protein, molecule) training examples.

The workflow of the CPDP framework is shown in Figure 5. CPDP establishes a bidirectional alignment mapping relationship between protein target representation and molecule representation derived from different representation models.

Using actual DTIs from BioHNs as constraints, we treat the alignment process as a contrastive learning task, where associated protein–molecule pairs are positive samples, and others are negative samples.

Specifically, given a batch of *N* proteins and *N* molecules, where the *i*th protein is associated with the *i*th molecule in actual DTIs, this protein–molecule pair is treated as a positive sample. As shown in the batch matrix in Figure 5, the diagonal elements of the matrix represent the positive samples, while the off-diagonal elements represent the negative samples. The training goal of CPDP is to predict the likelihood scores of actual associations for all possible *N* × *N* (protein, molecule) pairs in a batch.

To achieve this, we first utilize pre-trained biomedical representation models to map proteins and molecules to their respective representation spaces (see Figure 5a,b). Next, CPDP employs projection layers to map these separate representation spaces into a shared embedding space. In this unified space, CPDP aims to maximize the distance between the actual protein–molecule pairs in the batch, as measured by cosine similarity.

**Figure 6:**
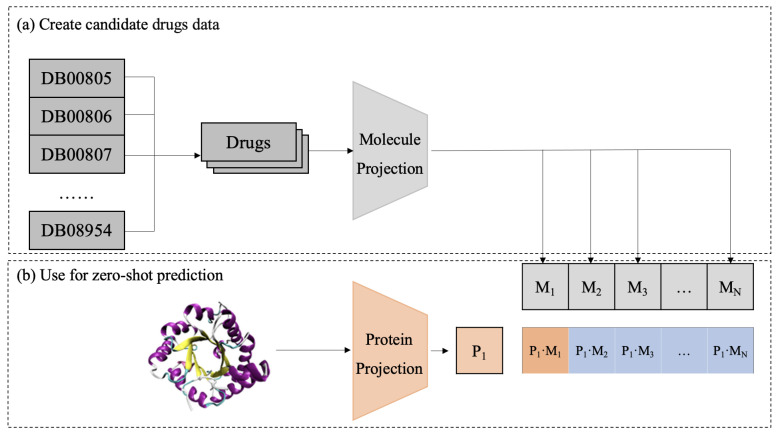
Diagram of the CPDP framework validation process.

In the test phase, a zero-shot approach is used to predict potential DTIs as in Figure 6. First, candidate drugs are extracted from a database and mapped into a shared embedding space using a pretrained molecular projection module. Then, the target protein is processed through a representation model and a protein projection module to obtain its embedding $P_{1}$. By computing cosine similarity between the protein and all candidate drugs $M_{1},M_{2},\cdots ,M_{n}$, CPDP predicts the likelihood of interaction between the target protein and each candidate drug.

### 3.3. More Details for CPDP

To mitigate over-fitting, we employ a weak labeling approach wherein each independent sample was considered a distinct category, even if multiple instances of the same protein or more than one related drug may appear within a batch. For a batch of sample pairs $S=\left\{\left(p_{1},m_{1}\right),\left(p_{1},m_{2}\right),\cdots ,\left(p_{i},m_{i}\right),\left(p_{i},m_{i+1}\right),\cdots \left(p_{N},m_{N}\right)\right\}$, where $p_{i}$ represents the *i*th protein and $m_{j}$ represents the *j*th drug molecule, the label is defined as follows:

(5) $$y_{ij}=\left\{\begin{array}{l} 1,\;\;\;\;\;\;\;\mathrm{if}\;i=j\\ no\;constraint,\;\;\;\mathrm{if}\;i\neq j \end{array}\right.$$

That is, for each pair $\left(p_{i},m_{i}\right)$, the label is set to 1, ignoring $\left(p_{i},m_{j}\right)\left(i\neq j\right)$ potential association labels within this batch. By not explicitly specifying interactions for the remaining pairs, CPDP is guided to focus on real protein–molecule interactions while effectively preserving interaction information in many-to-many DTIs scenarios.

The association likelihood between each protein–molecule pair is measured using cosine similarity and converted into probability scores through softmax.

During the validation phase, CPDP ranks the probability scores and selects one or more highly relevant molecules for the target protein. This approach allows for evaluating both the prediction accuracy and recall of CPDP.

During the experimental phase, we employ a bidirectional cross-entropy Loss, considering both the likelihood score $P_{i,j}$ of a protein matching a molecule and vice versa $Q_{j,i}$. The loss function consists of two parts:

(6) $$\begin{array}{c} L_{P\to M}=-\frac{1}{N}\sum_{i=1}^{N}\log P_{i,i}\\ L_{M\to P}=-\frac{1}{N}\sum_{i=1}^{N}\log Q_{i,i}\\ L=\frac{1}{2}\left(L_{P\to M}+L_{M\to P}\right) \end{array}$$

Here, $L_{P\to M}$ optimizes the association likelihood score when using a protein as the query, while $L_{M\to P}$ optimizes the association likelihood score when using a molecule as the query. This ensures the model learns stable feature representations from both perspectives.

## 4. Conclusions

We explore the potential of generalizing network-based DTI prediction to unseen biological entities. Building on existing biological representation models, we propose CPDP, a novel alignment framework for biomedical applications. By leveraging contrastive learning, we bridge associations between different biological entities (e.g., drugs and proteins) and use geometric distances to predict DTI likelihood scores, expanding the scope of drug screening and target discovery.

Inspired by the zero-shot concept, CPDP predicts associations between unseen biological entities for drug discovery and repurposing. It bypasses GNN limitations by generalizing DTI prediction via structural or textual representations without prior network associations. Compared to LLMs, CPDP enhances accuracy and adaptability through multimodal alignment.

In theory, CPDP can be extended to other biomolecular interactions, given informative representations and interaction data. However, this would require designing new alignment modules for each specific interaction type.

Despite these promising results, there is still room for improvement. Inspired by LLMs’ scale and zero-shot capabilities, incorporating larger biological datasets could expose the model to more types of biological entities, potentially driving the model toward a more generalizable direction. Since medical networks have sparse associations, future research could use self-supervised learning and domain adaptation to improve the model’s adaptability to sparse data.

## Figures and Tables

**Table 1:** Comparison of the performance of CPDP and Llama in predicting DTIs for novel drug identification (*N* = 4).

Protein–Molecules CPDP	Top-1	Top-3
MSSL2drug-JTVAE CPDP	**0.407**	**0.756**
MSSL2drug-Llama CPDP	0.393	0.708
MSSL2drug-BioGPT CPDP	0.282	0.691
Llama-7B prompt1	0.327	\
Llama-7B prompt2	0.327	\

Evaluation metrics include Top-1 and Top-3 accuracies.

**Table 2:** Comparison of the performance of CPDP and Llama in predicting DTIs for novel drug identification (*N* = 9).

Protein–Molecules CPDP	Top-1	Top-3	Top-5
MSSL2drug-JTVAE CPDP	**0.256**	**0.512**	**0.674**
MSSL2drug-Llama CPDP	0.225	0.517	0.663
MSSL2drug-BioGPT CPDP	0.145	0.390	0.597
Llama-7B prompt1	0.169	\	\
Llama-7B prompt2	0.011 *	\	\

Evaluation metrics include Top-1, Top-3, and Top-5 accuracies. * indicates most of Llama-7B queries failed to recognize the question or required more real-world evidence.

**Table 3:** Top-10 human protein targets ranked by CPDP predicted likelihood scores, along with supporting evidence.

Human Protein Target	Prediction	Likelihood Scores	Evidence
PTGFR	Travoprost	0.582	[27]
ACE	Ramipril	0.546	[28]
DPP4	Sitagliptin	0.532	[29]
AR	Methsuximide	0.505	[30]
ACHE	Tacrine	0.472	[31]
DHFR	Trimetrexate	0.420	[32]
HMGCR	Cerivastatin	0.376	[33]
CYP19A1	Exemestane	0.367	[34]
FDPS	Pamidronic Acid	0.367	[35]
AGTR1	Eprosartan	0.361	[36]

**Table 4:** Comparison of CPDP and Llama-7B query performance for drug reposition task (*N* = 4).

Protein–Molecules CPDP	Top-1	Top-3
Esm2-JTVAE CPDP	**0.798**	**0.938**
MSSL2drug-JTVAE CPDP	0.787	0.936
Llama-JTVAE CPDP	0.711	0.886
BioGPT-JTVAE CPDP	0.631	0.858
Llama-7B prompt1	0.456	\
Llama-7B prompt2	0.309	\

Evaluation metrics include Top-1 and Top-3 accuracies.

**Table 5:** Comparison of CPDP and Llama-7B query performance for drug reposition task (*N* = 9).

Protein–Molecules CPDP	Top-1	Top-3	Top-5
Esm2-JTVAE CPDP	**0.737**	**0.875**	**0.925**
MSSL2drug-JTVAE CPDP	0.721	0.860	0.914
Llama-JTVAE CPDP	0.639	0.797	0.860
BioGPT-JTVAE CPDP	0.561	0.709	0.821
Llama-7B prompt1	0.406	\	\
Llama-7B prompt2	0.250	\	\

Evaluation metrics include Top-1, Top-3, and Top-5 accuracies.

**Table 6:** The numbers of nodes and edges in the constructed BioHNs.

**Type of Node**	**Total**	**Train Set**	**Valid Set**	**Test Set**
Drug	670	603	67	125 *
Protein	1894	1512	382	86 *
Disease	731	\	\	\
**Type of edge**				
Drug-Protein Interactions	4839	4325	514	156 *
Drug–Disease Interactions	1103	\	\	\

* indicates incremental data that is not included in BioHNs.

## Data Availability

Source data for plots, raw data for counts and intensity measurements, and uncropped gel images generated in this study were submitted to the journal and are available from the corresponding authors upon request.

## Abbreviations

The following abbreviations are used in this manuscript:

DTIs	Drug–Target Interactions
GNN	Graph Neural Network
BioHNs	Biomedical Heterogeneous Network
LLMs	Large Language Models
TTD	Therapeutic Target Database
DDIs	Drug–Disease Interactions
SMILES	Simplified Molecular Input Line Entry System
AUPR	Area Under the Precision–Recall Curve
AUROC	Area Under the Receiver Operating Characteristic Curve
AP	Average Precision
MAP	Mean Average Precision
AR	Average Recall
MAR	Mean Average Recall
